# Impact of Epstein‐Barr Virus Donor Serostatus on Post‐Transplant Mortality and Post‐Transplant Lymphoproliferative Disorder in Epstein‐Barr Virus Seropositive Thoracic Organ Transplant Recipients: Data from the Organ Procurement and Transplantation Network

**DOI:** 10.1002/jmv.71117

**Published:** 2026-08-24

**Authors:** Khuloud Aldhaheri, Robert C. Wright, Stephen Lee, Mohsin Ali, Brian Clarke, Céline Bergeron, Allison Mah, Alissa Wright, Sara Belga

**Affiliations:** ^1^ Department of Internal Medicine, College of Medicine and Health Sciences United Arab Emirates University Al Ain United Arab Emirates; ^2^ Department of Medicine, Division of Infectious Disease University of British Columbia Vancouver British Columbia Canada; ^3^ Faculty of Pharmaceutical Sciences University of British Columbia Vancouver British Columbia Canada; ^4^ BC Children's Hospital Research Institute Vancouver British Columbia Canada; ^5^ Department of Medicine, Division of Infectious Diseases University of Saskatchewan Saskatoon Saskatchewan Canada; ^6^ Department of Pediatrics Division of Infectious Diseases, Hospital for Sick Children Toronto Ontario Canada; ^7^ Department of Pediatrics London Health Sciences Centre, Division of Infectious Diseases London Ontario Canada; ^8^ Department of Medicine, Division of Cardiology University of British Columbia Vancouver British Columbia Canada; ^9^ Department of Medicine, Division of Respiratory Medicine University of British Columbia Vancouver British Columbia Canada; ^10^ Immunity and Infection Research Centre, Vancouver Coastal Health Research Institute Vancouver British Columbia Canada

**Keywords:** Epstein‐Barr virus, heart transplant, Lung transplant, post‐transplant lymphoproliferative disorder

## Abstract

Donor Epstein‐Barr virus (EBV)‐seropositive (D + ) serostatus is a risk factor for post‐transplant lymphoproliferative disorder (PTLD) in EBV‐seronegative recipients. The impact of donor EBV serostatus on mortality and PTLD in EBV‐seropositive (R + ) thoracic organ transplant recipients is unknown. We analyzed 49 458 EBV R+ thoracic transplant recipients from the Organ Procurement and Transplantation Network (2004–2021). The primary exposure was EBV D+ serostatus; outcomes were death and PTLD. Associations were assessed using multivariable Cox and logistic regression models. EBV D+ serostatus was not associated with mortality but significantly reduced the hazard of PTLD in lung and heart‐lung (L/HLT) recipients (adjusted hazard ratio, 0.59; 95% CI: 0.42–0.83). No association was observed in heart transplants. Notably, 48.7% of PTLD cases in EBV R + L/HLT occurred early after transplant when the donor was EBV‐seronegative (D‐). In an exploratory subgroup analysis of PTLD cases, EBV D+ serostatus was associated with lower odds of early vs late PTLD (adjusted odds ratio [aOR]: 0.40; 95% CI: 0.22–0.73). Conversely, L/HLT had twice the odds of early PTLD (aOR: 2.62; 95% CI: 1.68 – 4.08). EBV D‐/R + L/HLT recipients carry a substantially higher risk of PTLD than EBV D + /R+ recipients, driven predominantly by early‐onset disease.

**Abbreviations**
aHR: adjusted hazard ratio
aOR: adjusted odds ratio
ATG: anti‐thymocyte globulin
cHR: crude hazard ratio
CI: confidence interval
CMV: cytomegalovirus
D: donor serostatus
D‐: donor‐seronegative
D/R: donor/recipient
D‐/R + : donor‐seronegative/recipient‐seropositive
D + : donor‐seropositive
D + /R‐: donor‐seropositive/recipient‐seronegative
D + /R + : donor‐seropositive/recipient‐seropositive
EBV: Epstein‐Barr virus
HLA: human leukocyte antigen
HLT: heart‐lung transplant
HR: hazard ratio
HT: heart transplant
IFN‐γ: interferon‐gamma
IgG: immunoglobulin G
LT: lung transplant
L/HLT: lung and heart‐lung transplant
OPTN: Organ Procurement and Transplantation Network
PTLD: post‐transplant lymphoproliferative disorder
R‐: recipient‐seronegative
R + : recipient‐seropositive
SOT: solid organ transplant
*T*_RM_
: tissue‐resident memory *T* cells
UNOS: United Network for Organ Sharing
VCA: viral‐capsid antigen
VIF: variance inflation factor

## 1 Introduction

Epstein‐Barr virus (EBV) infects over 90% of adults worldwide [1]. As a result, most adult donors and recipients are EBV‐seropositive at the time of transplant [2, 3, 4]. EBV establishes lifelong latency in memory *B* cells and evades immune recognition through restricted viral gene expression [1, 5, 6]. Post‐transplant EBV infection may arise from either a primary infection acquired from an EBV‐seropositive donor (D + ) or the reactivation of a previously established latent infection in EBV‐seropositive recipients (R + ) [7]. In solid organ transplant (SOT), immunosuppressive therapy impairs EBV‐specific *T* cell surveillance, which can lead to uncontrolled expansion of EBV‐infected *B* cells [1, 7, 8]. Clinically, post‐transplant EBV infection can present as asymptomatic EBV DNAemia, infectious mononucleosis syndrome, end‐organ disease, or post‐transplant lymphoproliferative disorder (PTLD) [7]. Current guidelines recommend determining EBV serostatus for all transplant candidates and donors [7]. In EBV R+ recipients, routine post‐transplant surveillance polymerase chain reaction testing is generally not recommended [3, 7].

The most concerning presentation of EBV in SOT recipients is PTLD, a heterogeneous disease of benign and malignant entities [9]. PTLD is the second most common malignancy among SOT recipients after non‐melanoma skin cancers [10]. Historically, mortality rates have ranged from 50% to 70%; however, contemporary data demonstrate improved prognosis with sequential immunochemotherapy [11, 12, 13]. Common risk factors include primary EBV infection in EBV donor‐seropositive/recipient‐seronegative (D + /R‐) recipients, advanced age, acute rejection, cytomegalovirus (CMV) co‐infection, *T*‐cell depleting induction therapy, net state of immunosuppression, white race, and, in heart transplant (HT), male sex [4, 10, 14, 15, 16]. Thoracic organ transplant recipients have a higher incidence of PTLD compared to other SOT, with lung transplant (LT) carrying the greatest risk [15, 17]. This increased risk is due to the higher net state of immunosuppression, and, in lung and heart‐lung transplant (L/HLT), the mass of donor lymphoid tissue transferred within the allograft [3, 7]. Additional factors among LT recipients include idiopathic pulmonary fibrosis and alemtuzumab induction [2, 18]. Among L/HLT recipients, nearly half of PTLD cases occur within the first year after transplant; this trend has been closely linked to EBV D + /R‐ serostatus and alemtuzumab induction therapy [17]. The vast majority of early PTLD cases are EBV‐positive compared to late PTLD, which supports EBV‐targeted surveillance and preemptive strategies in high‐risk recipients in the early post‐transplant period [7, 15, 17].

Although EBV D + /R‐ recipients carry the highest relative risk, the majority of adult thoracic transplant recipients are EBV R+ and thus represent the absolute burden of PTLD cases within this cohort [3, 4, 19, 20]. Despite the clinical relevance of this population, limited data exist on how EBV donor serostatus (D) impacts the risk of PTLD and mortality among EBV R+ thoracic transplant recipients. In this study, we hypothesize that EBV D+ serostatus influences mortality and the risk of PTLD among EBV R+ thoracic transplant recipients. The primary aims were to investigate the association between EBV D+ serostatus and post‐transplant mortality and PTLD, and to identify independent factors associated with PTLD risk in this population.

## 2 Materials and Methods

### 2.1 Study Design and Population

We conducted an observational cohort study using data from the United Network for Organ Sharing (UNOS)/Organ Procurement and Transplantation Network (OPTN), which prospectively collects information on all organ donors, transplant candidates, and recipients in the United States. The study cohort included EBV R+ recipients of thoracic transplants. Among 143 898 candidates, 84 313 received an HT, LT, or HLT during the study period between January 1, 2004, and December 31, 2021. From this cohort of 84 313 recipients, we sequentially excluded a total of 34 855 recipients based on the following criteria: age <18 years (*n* = 8023), prior transplant (*n* = 2835), multiorgan transplant other than HLT (*n* = 2487), missing or non‐interpretable donor EBV viral‐capsid antigen (VCA) immunoglobulin G (IgG) [*n* = 10 741], and missing, non‐interpretable, or negative recipient EBV VCA‐IgG (*n* = 10 769). The OPTN coding for EBV‐VCA IgG serostatus and the distribution of non‐interpretable results are provided in the Material S1.

**Figure 1:**
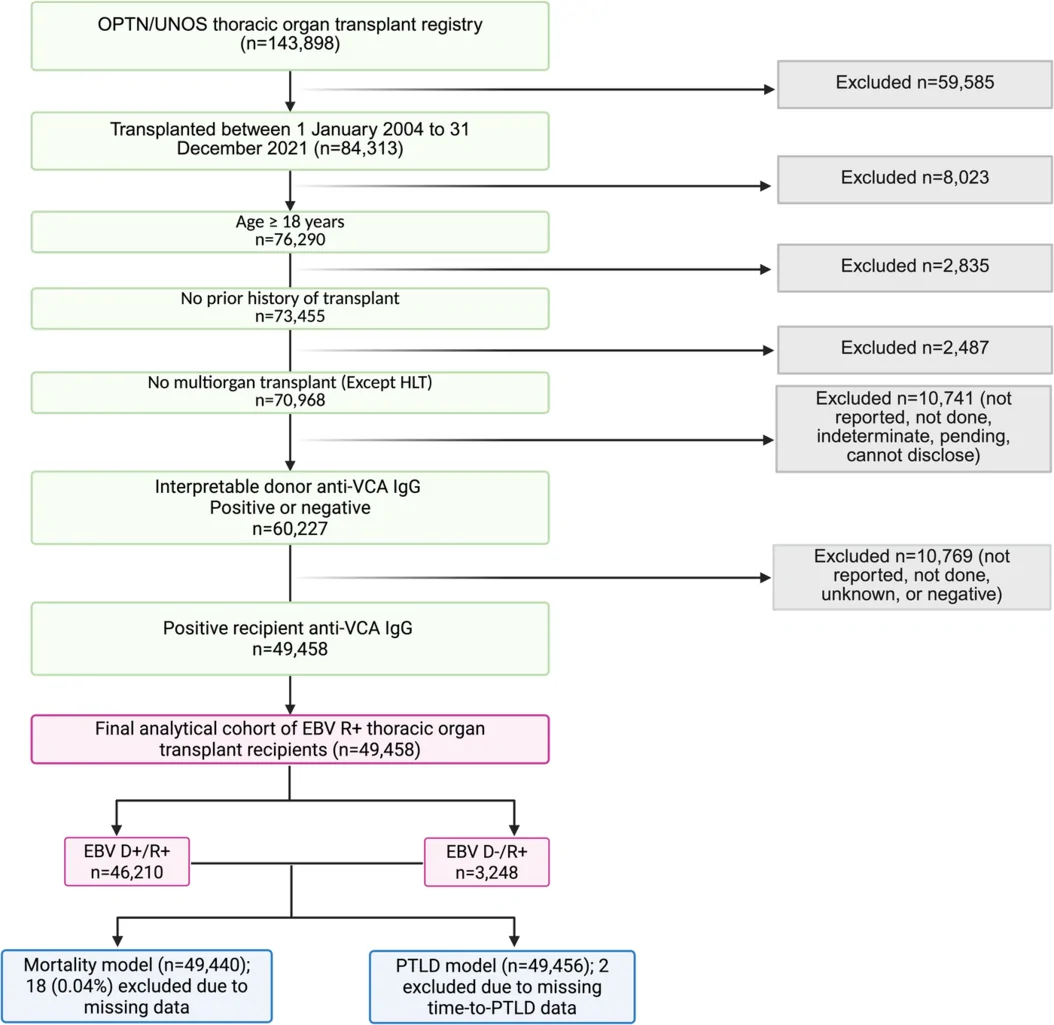
Cohort selection and derivation of the analytic population of EBV R+ thoracic organ transplant recipients. D‐/R + , donor‐seronegative/recipient‐seropositive; D + /R + , donor‐seropositive/recipient‐seropositive; EBV, Epstein‐Barr virus; HLT, heart‐lung transplant; IgG, immunoglobulin G; OPTN, Organ Procurement and Transplantation Network; PTLD, post‐transplant lymphoproliferative disorder; R + , recipient‐seropositive; UNOS, United Network for Organ Sharing; VCA, viral capsid antibody. Created in BioRender. Aldhaheri, K. (2026) https://BioRender.com/3ywnrwn.

The final analytical cohort comprised 49 458 EBV R+ adult thoracic transplant recipients, of whom 46 210 (93.4%) were EBV donor‐seropositive/recipient‐seropositive (D + /R + ), and 3248 (6.6%) were EBV donor‐seronegative/recipient‐seropositive (D‐/R + ) [Figure 1]. Participants entered the study on the day of transplant and were followed until the occurrence of the specified primary outcome for each model (death or PTLD) or until censoring. Censoring was defined as loss to follow‐up or administrative censoring on October 6, 2023. Due to their shared immunobiology, L/HLT recipients were grouped for analysis, whereas HT recipients were evaluated separately to allow organ‐stratified comparisons. Additional complete‐case exclusions due to missing covariate data in the mortality model (*n* = 18) and the PTLD model (*n* = 2) were negligible.

### 2.2 Exposure

The primary exposure was the EBV D+ serostatus at the time of organ procurement, determined by anti‐VCA IgG testing and recorded as a single qualitative result for each donor in the OPTN database. The specific assay manufacturer or laboratory methodology utilized by individual transplant centres is not captured in the registry.

### 2.3 Covariates

Covariates were selected a priori based on biological plausibility and established associations with mortality or PTLD risk. Definitions were obtained from the OPTN data dictionary fields as follows: 1) Demographic variables included transplant type (HT, LT, HLT); donor and recipient age (continuous, in years); recipient sex (as recorded on the transplant centres’ electronic data forms); recipient race and ethnicity (combined into a single, mutually exclusive variable based on data provided by transplant centres: non‐Hispanic white, non‐Hispanic black, Hispanic or Latinx, and other); and insurance type at the time of transplant (Medicaid, Medicare, private); 2) Clinical variables included total waitlist time (years); CMV donor/recipient (D/R) serostatus; mechanical assistance at the time of transplant (extracorporeal membrane oxygenation or mechanical ventilation); prior malignancy (any recipient malignancy before transplant); and early treated rejection (defined as any treated rejection episode during the index transplant admission); 3) Immunosuppression, included induction immunosuppression (categorized by pharmacological class, excluding corticosteroids) and maintenance immunosuppression at the time of discharge from the index transplant admission. Triple maintenance immunosuppression was defined as the concurrent use of three of the following: calcineurin inhibitor, antimetabolite, mTOR inhibitor, and corticosteroids at the time of discharge. A comprehensive list of covariate definitions is provided in the Material S1. Lastly, transplant indications were extracted for descriptive purposes but were not included in the analyses.

### 2.4 Outcomes

The primary outcomes were all‐cause mortality and PTLD, as captured in OPTN registry malignancy forms. Early PTLD, defined as PTLD occurring within the first year (≤365 days) after transplant, was evaluated as a secondary outcome.

### 2.5 Statistical Analysis

Baseline characteristics are presented descriptively as counts and proportions. Cox proportional hazards models were used to estimate the associations between EBV D+ serostatus and mortality or PTLD, with results reported as hazard ratios (HRs) and 95% confidence intervals (CIs). In the PTLD model, competing‐risks regression was initially considered to account for the competing event of death; however, because death was not identified as a competing risk that would affect hazard estimates, the standard Cox regression was used. Survival curves were visualized using Kaplan‐Meier estimates, and comparisons were performed using the log‐rank test. Two‐sided *p*‐values < 0.05 were considered statistically significant. Multicollinearity was assessed by computing Variance Inflation Factors (VIFs) for each predictor, with values greater than 5 indicating potential multicollinearity.

Multivariable models were adjusted for specific covariates based on established clinical associations with post‐transplant mortality and PTLD risk. Both models included the following variables in the adjustment: recipient and donor age, recipient sex, recipient race, CMV D/R (CMV R+ and CMV D + /R‐), and early‐treated rejection [4, 21, 22, 23, 24]. To prevent violations of the proportional hazards assumption, transplant type (L/HLT vs HT) was incorporated as a stratification factor, allowing the hazard to vary by transplant type without assuming proportional hazards across groups. Insurance type, prior malignancy, and mechanical assistance at the time of transplant were included in the mortality model because of their established associations with post‐transplant survival [14, 25, 26]. These variables were excluded from the PTLD model, as they lack an established association with PTLD risk. Immunosuppression variables were evaluated in a separate sensitivity analysis due to substantial missing data in the OPTN registry.

The primary analyses used a complete‐case analysis approach, with missingness below 0.1% for covariates other than immunosuppression fields. Univariable and multivariable analyses were conducted on the same complete‐case cohort. To assess potential selection bias, we compared the baseline characteristics of the analytic cohort with those of the excluded cohort that had missing donor or recipient EBV serostatus data. Analyses were performed with Stata/SE (StataCorp LLC, version 19.5, College Station, TX).

### 2.6 Sensitivity Analyses

The primary Cox model was refitted to include, in separate models, a variable indicating receipt of any *T*‐cell‐depleting therapy and a variable indicating use of triple maintenance immunosuppression at the time of the index admission discharge. Both were complete‐case analyses conducted within the L/HLT and HT strata. Comprehensive methods of the sensitivity analyses are provided in the Material S1.

### 2.7 Exploratory Analyses

Donor‐recipient human leukocyte antigen (HLA) mismatch was assessed as a continuous variable across HLA‐A, HLA‐B, and HLA‐DR, and as a binary variable (low: 0–2 vs high: ≥3 mismatches) in multivariable Cox models stratified by L/HLT and HT. Logistic regression models, one including and the other excluding the variable for *T*‐cell depleting therapy, were constructed to examine factors associated with early vs late PTLD among a subgroup of EBV R+ thoracic transplant recipients diagnosed with PTLD. Comprehensive methods of the exploratory analyses are provided in the Material S1.

### 2.8 Ethics Statement

Ethics review was exempt as per the Government of Canada Tri‐Council Policy Statement 2, article 2.2.

## 3 Results

### 3.1 Baseline Characteristics

The median age of recipients was 59 years in both groups, and the majority were male (68.4% in EBV D‐/R + ; 66.9% in EBV D + /R + ). Donor age was younger in EBV D‐/R+ (median 24 years, IQR 18–35) compared to EBV D + /R+ (median 32 years, IQR: 23–44). The cohort comprised 48.3% LT (*n* = 23 910), 51.0% HT (*n* = 25 237), and 0.6% HLT (*n* = 311) recipients. Induction and maintenance immunosuppression were overall comparable between groups (Table 1). Characteristics of recipients excluded due to missing donor or recipient EBV serostatus are presented in Table S1.

**Table 1:** Baseline demographics and clinical characteristics of EBV R+ thoracic organ transplant recipients by EBV donor serostatus.

Factor *N* (%)	EBV D‐/R + *n* = 3248	EBV D + /R + *n* = 46 210
Age		
Donor, median	24.0 (18.0, 35.0)	32.0 (23.0, 44.0)
Recipient, median	59.0 (49.0, 65.0)	59.0 (50.0, 65.0)
Recipient, categorical (years)		
18–39	453 (13.9)	5810 (12.6)
40–59	1312 (40.4)	18884 (40.9)
≥60	1483 (45.7)	21516 (46.6)
Male	2222 (68.4)	30903 (66.9)
Race, categorical		
White	2378 (73.2)	33467 (72.4)
Black	496 (15.3)	7450 (16.1)
Hispanic or Latinx	262 (8.1)	3607 (7.8)
Other	112 (3.4)	1686 (3.6)
Insurance type		
Medicaid	335 (10.3)	4559 (9.9)
Medicare	1255 (38.6)	18055 (39.1)
Private	1536 (47.3)	21825 (47.2)
CMV D/R serostatus		
CMV D‐/R‐	845 (26.0)	7485 (16.2)
CMV D + /R‐	593 (18.3)	12037 (26.1)
CMV D + /R+	740 (22.8)	17111 (37.0)
CMV D‐/R+	1070 (32.9)	9577 (20.7)
Mechanical assistance at the time of transplant		
ECMO	92 (2.8)	1547 (3.3)
Ventilator	99 (3.0)	1534 (3.3)
Prior malignancy	266 (8.2)	3978 (8.6)
Time on transplant list, categorical (years)		
<1	2763 (85.1)	39447 (85.4)
1–3	384 (11.8)	5498 (11.9)
≥3	101 (3.1)	1265 (2.7)
Type of organ transplant		
Lung	1582 (48.7)	22328 (48.3)
Heart	1646 (50.7)	23591 (51.1)
Heart and lung	20 (0.6)	291 (0.6)
Indication for lung transplant		
COPD	375 (23.7)	4880 (21.9)
IPF	562 (35.5)	8260 (37.0)
CF	144 (9.1)	1855 (8.31)
Indication for heart transplant		
Dilated idiopathic cardiomyopathy	618 (37.6)	8548 (36.2)
Dilated ischemic cardiomyopathy	502 (30.5)	7398 (31.4)
Other dilated cardiomyopathy	292 (17.7)	4303 (18.2)
Indication for heart/lung transplant		
PH	6 (30.0)	111 (38.1)
IPF	1 (5.0)	28 (9.6)
Sarcoidosis	2 (10.0)	19 (6.5)
Early treated rejection	286 (8.8)	4175 (9.0)
Induction immunosuppression		
IL‐2 receptor antagonist	1430 (44.0)	20370 (44.1)
ATG	398 (12.3)	6365 (13.8)
Anti‐CD20	13 (0.40)	275 (0.60)
Anti‐CD52	103 (3.17)	1095 (2.37)
Other	21 (0.65)	251 (0.54)
Maintenance immunosuppression by agent		
Calcineurin inhibitors	3136 (96.6)	44530 (96.4)
Tacrolimus	2955 (91.0)	42074 (91.1)
Cyclosporine	202 (6.22)	2745 (5.94)
Antimetabolites	3092 (95.2)	43727 (94.6)
Mycophenolic acid	2857 (88.0)	40447 (87.5)
Azathioprine	250 (7.70)	3553 (7.69)
mTOR inhibitors	10 (0.31)	256 (0.55)
Corticosteroids	3038 (93.5)	43070 (93.2)
Other	23 (0.71)	330 (0.71)
Triple maintenance immunosuppression	2910 (89.6)	41296 (89.4)

*Note:* Median IQR for continuous variables and *n* (%) for categorical variables.

Abbreviations: R + : recipient‐seropositive; CF, cystic fibrosis; CMV, cytomegalovirus; COPD, chronic obstructive pulmonary disease; D‐/R + , donor‐seronegative/recipient‐seropositive; D + /R + , donor‐seropositive/recipient‐seropositive; D‐/R‐, donor‐seronegative/recipient‐seronegative; D + /R‐, donor‐seropositive/recipient‐seronegative; D/R, donor‐serostatus/recipient‐serostatus; EBV, Epstein‐Barr virus; ECMO, extracorporeal membrane oxygenation and ventilation; IPF, idiopathic pulmonary fibrosis; IQR, interquartile range; mTOR inhibitors, mechanistic target of rapamycin inhibitors; PH, pulmonary hypertension.

### 3.2 Mortality After Thoracic Organ Transplant

Median recipient survival was 9.14 years (IQR: 3.67–15.5); 6.42 years (IQR: 2.55–11.4) for L/HLT recipients and 12.8 years (IQR: 6.10–18.1) for HT recipients. Following L/HLT, the incidence rate of death was 11.5 per 100 person‐years in EBV D + /R+ and 11.1 per 100 person‐years in EBV D‐/R+ (*p* = 0.355). Conversely, after HT, the mortality rate was 5.3 per 100 person‐years in EBV D + /R+ and 5.0 per 100 person‐years in EBV D‐/R+ (*p* = 0.195).

**Figure 2:**
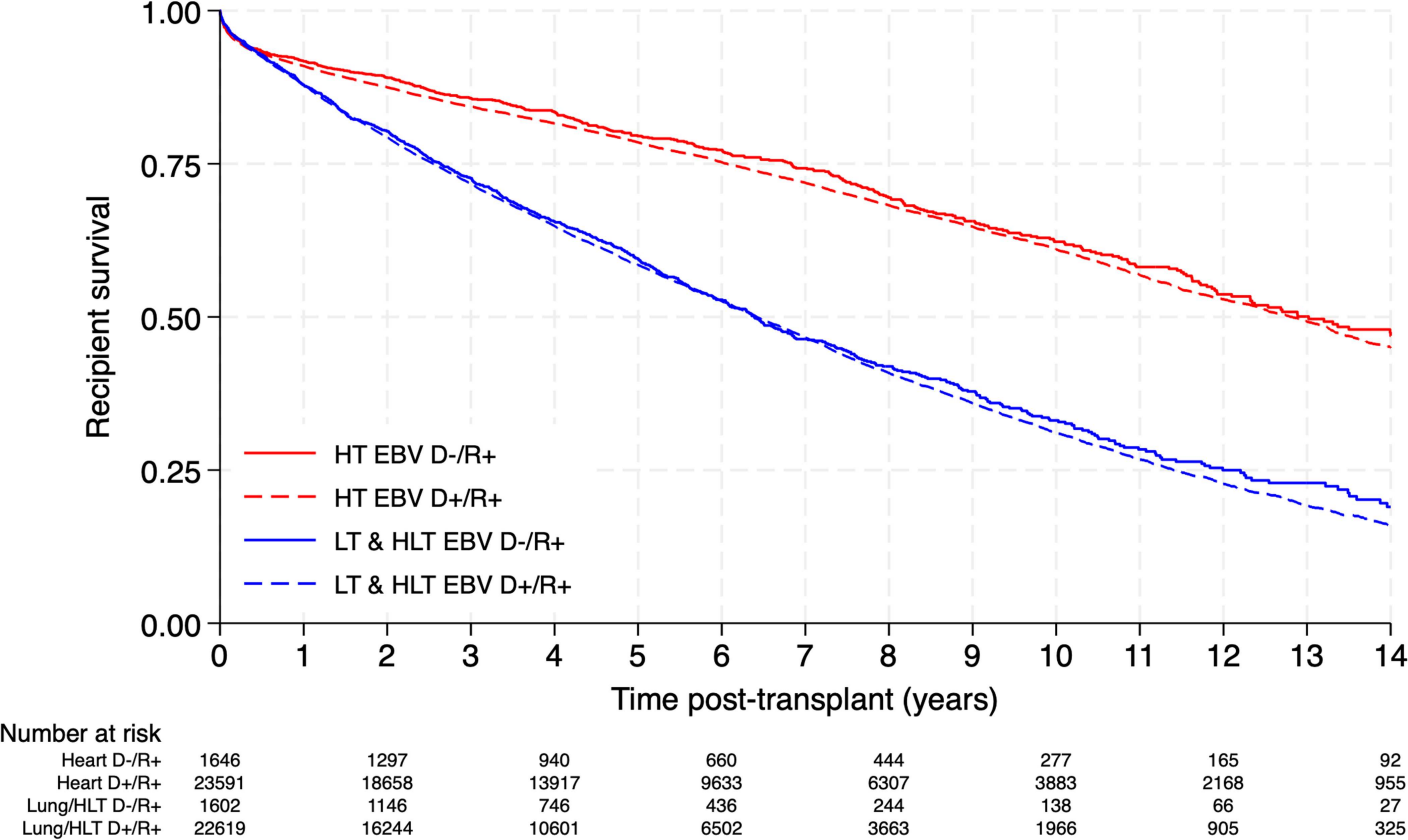
Kaplan‐Meier survival plots for EBV D‐/R+ versus EBV D + /R+ in lung and heart‐lung transplant recipients compared to heart transplant recipients. D + /R + , donor‐seropositive/recipient‐seropositive; D‐/R + , donor‐seronegative/recipient‐seropositive; EBV, Epstein‐Barr virus; HLT, heart‐lung transplant; HT, heart transplant; LT, lung transplant.

In L/HLT, EBV D+ status was not associated with increased hazard of death (crude hazard ratio [cHR]: 1.04; 95% CI: 0.964–1.113; adjusted HR [aHR]: 0.98; 95% CI: 0.912–1.054) [Table 2, Figure 2]. Factors independently associated with increased hazard of death were donor age (aHR: 1.004; 95% CI: 1.003–1.006), and the following recipient factors: age (aHR: 1.02; 95% CI: 1.018–1.022), public insurance (Medicaid [aHR: 1.27; 95% CI: 1.130–1.432], and Medicare [aHR: 1.15; 95% CI: 1.039–1.269]), pre‐transplant mechanical assistance (aHR: 1.34; 95% CI: 1.254–1.441), pre‐transplant malignancy (aHR: 1.12; 95% CI: 1.050–1.187), CMV D + /R‐ (aHR: 1.28; 95% CI: 1.212–1.356), CMV R+ (aHR: 1.15; 95% CI: 1.091–1.209) and early treated rejection within index transplant admission (aHR: 1.32; 95% CI: 1.234–1.406). Female sex was associated with decreased hazard of death (aHR: 0.93; 95% CI: 0.894–0.962).

**Table 2:** Univariable and multivariable Cox regression analyses for mortality in lung, heart‐lung, and heart transplantation in EBV R+ recipients.

	Lung and heart‐lung transplant		Heart transplant	
	Crude HR (95% CI)	Adjusted HR (95% CI)	Crude HR (95% CI)	Adjusted HR (95% CI)
EBV (D + )	1.04 (0.964–1.113)	0.98 (0.912–1.054)	1.08 (0.982–1.183)	1.00 (0.911–1.100)
Donor age	1.01 (1.004–1.007)	1.004 (1.003–1.006)	1.01 (1.010–1.014)	1.01 (1.008–1.012)
Recipient age	1.02 (1.019–1.022)	1.02 (1.018–1.022)	1.01 (1.010–1.013)	1.01 (1.007–1.012)
Female sex	0.89 (0.861–0.925)	0.93 (0.894–0.962)	0.92 (0.875–0.972)	0.92 (0.869–0.967)
Race				
White	1.06 (1.016–1.113)	1.07 (0.955–1.205)	0.90 (0.857–0.941)	1.07 (0.952–1.213)
Black	0.98 (0.924–1.041)	1.11 (0.980–1.265)	1.20 (1.143–1.270)	1.30 (1.144–1.470)
Hispanic or Latinx recipients	0.91 (0.849–0.980)	1.01 (0.886–1.157)	0.95 (0.870–1.035)	1.06 (0.919–1.225)
Insurance				
Medicaid	0.99 (0.923–1.062)	1.27 (1.130–1.432)	1.12 (1.048–1.198)	1.21 (1.060–1.388)
Medicare	1.25 (1.207–1.295)	1.15 (1.039–1.269)	1.34 (1.277–1.399)	1.16 (1.026–1.316)
Private	0.82 (0.793–0.851)	1.02 (0.921–1.124)	0.73 (0.695–0.761)	0.89 (0.783–1.003)
CMV D/R serostatus				
CMV D + /R‐	1.11 (1.064–1.151)	1.28 (1.212–1.356)	1.01 (0.960–1.065)	1.17 (1.083–1.257)
CMV R+	1.02 (0.989 – 1.061)	1.15 (1.091 – 1.209)	1.09 (1.038 – 1.137)	1.13 (1.053 – 1.205)
Mechanical assistance at the time of transplant	1.16 (1.080–1.238)	1.34 (1.254–1.441)	1.31 (1.131–1.513)	1.44 (1.244–1.665)
Prior malignancy	1.24 (1.171–1.321)	1.12 (1.050–1.187)	1.10 (1.013–1.189)	1.07 (0.983–1.156)
Early treated rejection	1.26 (1.185–1.349)	1.32 (1.234–1.406)	1.05 (0.980–1.130)	1.09 (1.011–1.167)

Abbreviations: CI, confidence interval; CMV, cytomegalovirus; D + , donor‐seropositive; D + /R + , donor‐seropositive/recipient‐seropositive; D + /R‐, donor‐seropositive/recipient‐seronegative; D‐/R + , donor‐seronegative/recipient‐seropositive; D/R, donor‐serostatus/recipient‐serostatus; EBV, Epstein‐Barr virus; HR, hazard ratio; R + , recipient‐seropositive.

In HT, EBV D+ was not associated with mortality (cHR: 1.08; 95% CI: 0.98 – 1.18; aHR: 1.00; 95% CI: 0.911–1.100) [Table 2, Figure 2]. Factors independently associated with increased hazard of death included donor age (aHR: 1.01; 95% CI: 1.008–1.012), and the following recipient factors: age (aHR: 1.01; 95% CI: 1.008–1.012), non‐Hispanic black race (aHR: 1.30; 95% CI: 1.144–1.470), public insurance (Medicaid insurance [aHR: 1.21; 95% CI: 1.060–1.388], and Medicare [aHR: 1.16; 95% CI: 1.022–1.311]), pre‐transplant mechanical assistance (aHR: 1.44; 95% CI: 1.243–1.664), CMV D + /R‐ (aHR: 1.17; 95% CI: 1.083–1.1257), CMV R+ (aHR: 1.13; 95% CI: 1.053–1.205), and early treated rejection within index transplant admission (aHR: 1.09; 95% CI: 1.015–1.171). Female sex was associated with decreased hazard of death (aHR: 0.92; 95% CI: 0.876–0.975). Causes of death are presented in Table S2.

### 3.3 PTLD After Thoracic Organ Transplant

The incidence rate of PTLD in L/HLT was 0.32 per 100 person‐years in EBV D + /R+ compared to 0.55 per 100 person‐years in EBV D‐/R+ (*p* = 0.004). In HT, the incidence rate was 0.20 per 100 person‐years in EBV D + /R+ compared to 0.17 per 100 person‐years in EBV D‐/R+ (*p* = 0.488).

**Figure 3:**
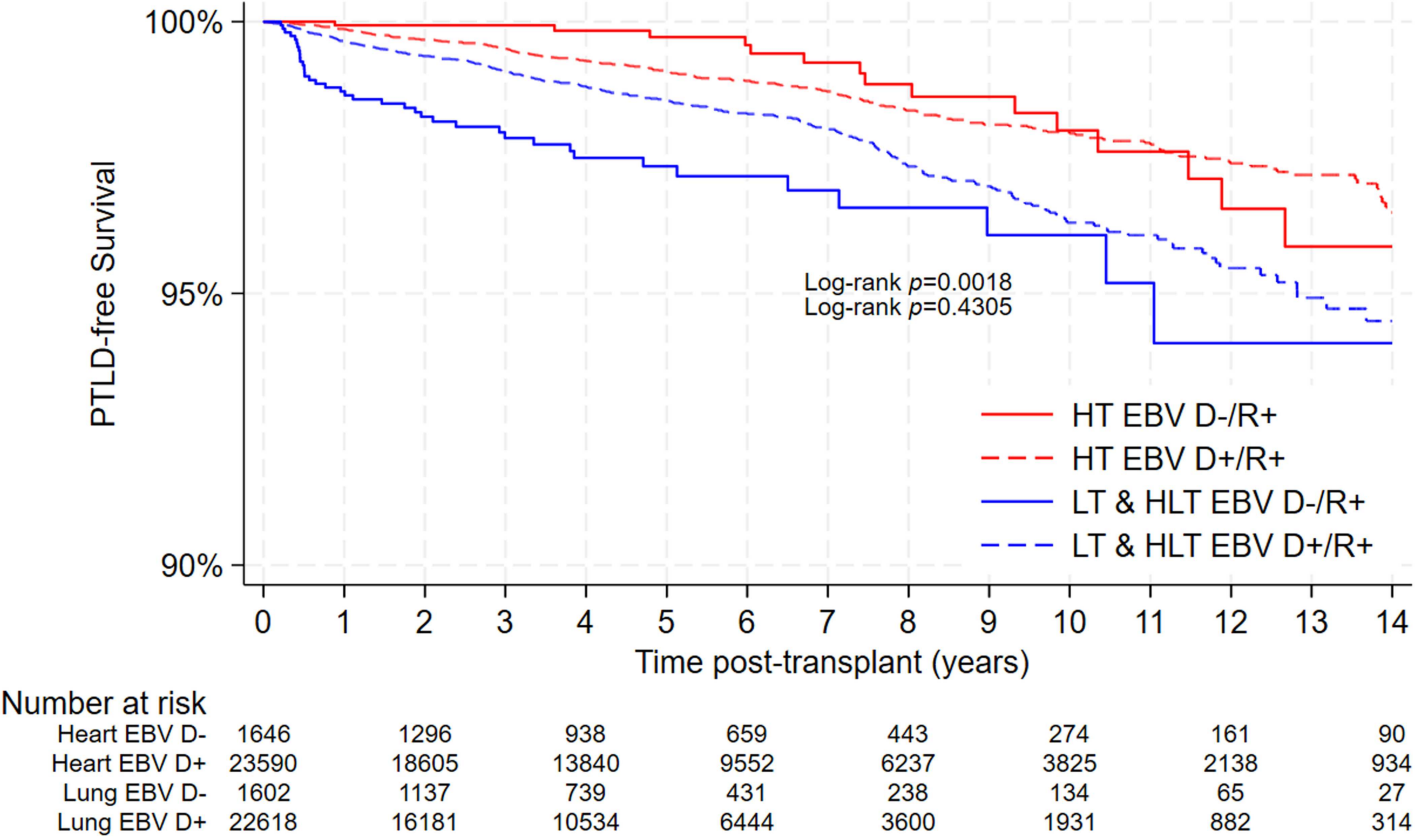
Kaplan‐Meier PTLD‐free survival plots for EBV D‐/R+ versus EBV D + /R+ in lung and heart‐lung transplant recipients compared to heart transplant recipients. D + /R + , donor‐seropositive/recipient‐seropositive; D‐/R + , donor‐seronegative/recipient‐seropositive; EBV, Epstein‐Barr virus; HLT, heart‐lung transplant; HT, heart transplant, LT, lung transplant; PTLD, post‐transplant lymphoproliferative disorder.

In L/HLT, EBV D+ status was associated with decreased hazard of PTLD in both univariable and multivariable analyses (cHR: 0.59; 95% CI: 0.426–0.827; aHR: 0.59; 95% CI: 0.424–0.831) [Table 3, Figure 3]. Recipient age was independently associated with increased hazard of PTLD (aHR: 1.02; 95% CI: 1.008–1.027), while female sex was protective (aHR: 0.73; 95% CI: 0.585–0.910).

**Table 3:** Univariable and multivariable Cox regression analyses for PTLD in lung, heart‐lung, and heart transplantation in EBV R+ recipients.

	Lung and heart‐lung transplant		Heart transplant	
	Crude HR (95% CI)	Adjusted HR (95% CI)	Crude HR (95% CI)	Adjusted HR (95% CI)
EBV (D + )	0.59 (0.426–0.827)	0.59 (0.424–0.831)	1.22 (0.739–2.028)	1.21 (0.726–2.007)
Donor age	1.00 (0.990–1.004)	1.00 (0.991–1.006)	1.00 (0.987–1.007)	0.99 (0.984–1.004)
Recipient age	1.02 (1.009–1.029)	1.02 (1.008–1.027)	1.02 (1.012–1.034)	1.02 (1.009–1.032)
Female	0.67 (0.543–0.838)	0.73 (0.585–0.910)	0.68 (0.507–0.910)	0.74 (0.549–0.996)
Race				
Black	0.64 (0.423–0.967)	0.67 (0.402–1.132)	0.66 (0.479–0.922)	1.12 (0.667–1.875)
White	1.24 (0.938–1.630)	0.94 (0.662–1.337)	1.65 (1.251–2.181)	1.57 (1.006–2.457)
CMV D/R serostatus				
CMV D + /R‐	1.15 (0.920–1.445)	1.19 (0.875–1.632)	1.29 (1.004–1.666)	1.29 (0.898–1.854)
CMV R+	0.91 (0.739–1.111)	1.02 (0.771–1.363)	0.85 (0.674–1.071)	1.07 (0.760–1.495)
Early treated rejection	1.32 (0.907–1.913)	1.41 (0.971–2.052)	1.08 (0.753–1.555)	1.20 (0.836–1.735)

Abbreviations: CI, confidence interval; CMV, cytomegalovirus; D + , donor‐seropositive; D + /R‐, donor‐seropositive/recipient‐seronegative; D/R, donor‐serostatus/recipient‐serostatus; EBV, Epstein‐Barr virus; HR, hazard ratio; PTLD, post‐transplant lymphoproliferative disorder; R + , recipient‐seropositive.

#### 3.3.1 HT

EBV D+ status was not associated with the hazard of PTLD (cHR: 1.22; 95% CI: 0.739–2.028; aHR: 1.21; 95% CI: 0.726–2.007). Older recipient age and non‐Hispanic white race were each associated with increased hazard of PTLD (aHR: 1.02; 95% CI: 1.009–1.032, and 1.57; 95% CI: 1.006–2.457, respectively). Female sex was again protective (aHR: 0.74; 95% CI: 0.549–0.996). No collinearity was identified (mean VIF = 1.46; all < 2.5).

### 3.4 Sensitivity Analyses

In L/HLT recipients, additional adjustment for *T*‐cell depletion therapy (aHR: 0.46; 95% CI: 0.313–0.681) and triple maintenance immunosuppression (aHR: 0.59; 95% CI: 0.421–0.825) did not alter the association between EBV D+ serostatus and PTLD (Table S3). Neither was independently associated with PTLD, and the HT results remained null in both models.

### 3.5 Exploratory Analyses

Donor‐recipient HLA mismatch, modelled as a continuous or binary variable, was not associated with PTLD risk in either L/HLT or HT (Table S4). The association between EBV D+ and lower PTLD risk in L/HLT remained unchanged after HLA adjustment.

Among L/HLT recipients who developed PTLD, early PTLD occurred in 48.7% of the EBV D‐/R+ group and 21.9% of the EBV D + /R+ group (Table S5). In logistic regression restricted to PTLD cases, EBV D+ serostatus was associated with lower odds of early PTLD (adjusted odds ratio [aOR]: 0.40; 95% CI: 0.22–0.73), and L/HLT was associated with increased odds of early PTLD (aOR: 2.62; 95% CI: 1.68–4.08), while *T*‐cell depletion was not associated with early PTLD (Table S6).

## 4 Discussion

In this registry‐based study of 49,458 EBV R+ thoracic transplant recipients, EBV D+ serostatus was associated with a reduced risk of PTLD among L/HLT recipients. Exploratory analyses suggest that this protective effect is mainly driven by a reduction in early PTLD risk. Conversely, EBV D+ serostatus was not associated with PTLD risk in HT, or with post‐transplant mortality in EBV R+ thoracic transplant recipients.

Our findings contribute to the limited literature regarding EBV R+ thoracic transplant recipients by identifying EBV D+ serostatus as a potential protective factor against PTLD within the EBV R + L/HLT population. Beyond EBV D serostatus, our multivariable models confirmed that advanced recipient age was independently associated with increased risk of PTLD, consistent with prior literature [10, 18]. Additionally, multivariable analysis demonstrated that female sex was independently associated with reduced PTLD risk in both L/HLT and HT recipients, providing clarity on previous conflicting reports on male sex as a risk factor [2, 5, 10, 18]. We observed an independent association between non‐Hispanic white recipient race and an increased risk of PTLD among HT recipients, corroborating similar trends reported in earlier registry‐based studies [4, 16]. Furthermore, our finding of no association between CMV D + /R‐ and R+ with PTLD is consistent with recent registry analyses and several large contemporary studies, which also found no association [18, 27]. This suggests that CMV co‐infection may become a significant risk factor only when combined with specific other high‐risk conditions.

The allograft‐specific nature of the EBV D+ protective effect is consistent with the distinctive immunobiology of lung allografts. Courtwright et al. previously reported a lower crude incidence of PTLD among EBV D + /R+ compared with EBV D‐/R + LT recipients (1.3% vs 2.4%, respectively), suggesting a possible protective effect of donor allograft EBV immunity [4]. It remains unknown whether this difference is driven by allograft‐localized disease or by the protective effects of EBV D+ serostatus against PTLD within the graft; previous studies have not reported PTLD locations based on donor serostatus in EBV R+ recipients, and our analysis of the UNOS/OPTN registry was limited by the lack of data fields that document PTLD location [11, 28, 29]. Recent studies demonstrate that donor‐derived tissue‐resident memory *T* cells (*T*_RM_) persist within the lung allograft for at least 15 months post‐transplant, co‐localize with the bronchus‐associated lymphoid tissue, retain functional capacity ex vivo, and produce effector cytokines, including interferon‐gamma (IFN‐γ) [30]. Mechanistic data further established that *T*_RM_‐derived IFN‐γ orchestrates subset‐specific antiviral programming in neighbouring airway epithelial cells, providing a direct mechanism to restrict viral propagation at the mucosal surface [31]. Moreover, donor‐derived EBV‐specific T_RM_ can account for up to 12% of all CD8^+^ *T* cells in the lung allograft at the time of transplant [31]. We hypothesize that in the context of an EBV D+ allograft, donor‐derived EBV‐specific *T*_RM_ cells might locally control EBV replication during the initial period of intense immunosuppression. In EBV D‐ allografts, the absence of pre‐existing donor‐derived EBV‐specific *T*_RM_ cells would delay this response, and EBV infection of passenger donor *B* cells is likely to occur in the absence of any donor‐derived EBV‐specific *T*_RM_ [30].

Our pre‐specified sensitivity analysis of recipients with complete immunosuppression data, specifically *T*‐cell depletion and triple maintenance immunosuppression, demonstrated a persistent protective association of EBV D+ serostatus within the L/HLT cohort after adjustment. These results suggest that the protective effect of EBV D+ serostatus is independent of baseline immunosuppression and receipt of *T*‐cell depleting therapy [27]. This finding aligns with the broader literature, which has not identified a consistent association between any single immunosuppressive agent and PTLD risk [7, 18]. Instead, the cumulative burden of immunosuppression is implicated as the primary contributing factor [9, 10, 32]. Our analysis of cumulative immunosuppression was limited because the OPTN/UNOS registry does not capture agent‐specific dosing, drug levels, longitudinal changes to maintenance regimens, or cumulative drug exposures over time, which limited the depth of confounding adjustments that could be applied in this study [33].

Although these findings provide novel insights into PTLD risk stratification, prospective validation remains necessary before implementing immediate changes in clinical practice. The risk of PTLD observed in EBV D‐/R + L/HLT recipients is concentrated in the first year after transplant, a period during which current guidelines do not recommend EBV DNA surveillance for EBV R+ recipients [7]. Additionally, a recent single‐centre cohort study of EBV R + LT recipients demonstrated that EBV DNA monitoring in asymptomatic EBV R + LT recipients had limited predictive value for PTLD [3]. Our identification of a specific EBV D‐/R + L/HLT subgroup with a significantly higher risk of early PTLD prompts reconsideration of its classification. Unlike in EBV D + /R‐ recipients, where the association between EBV DNAemia and PTLD is well established, the patterns and levels of EBV DNAemia that predict PTLD in EBV D‐/R+ recipients remain unknown [7]. Therefore, defining these viral load kinetics represents a high‐priority area for prospective studies, and future research should evaluate whether this specific subgroup would benefit from targeted surveillance during the first year post‐transplant.

The lack of association between EBV *D* serostatus and mortality may be partly explained by the widespread adoption of rituximab‐based therapies, which have substantially improved PTLD survival outcomes. A single‐centre study demonstrated a trend towards improved 5‐year survival, with rituximab reaching approximately 51% compared with 26% in historical cohorts treated without rituximab [11]. Furthermore, prospective trials have established rituximab‐based sequential therapy as the standard treatment for CD20‐positive PTLD, with response rates approaching 90% [12, 13]. The reduction of immunosuppression is now universally recommended as the initial management step upon the diagnosis of PTLD, and when combined with these therapeutic regimens, it may effectively mitigate the mortality risk associated with PTLD and EBV *D* serostatus [5, 9, 10, 11, 34]. Notably, the observed relationship between EBV D‐/R+ in L/HLT recipients and early PTLD, which is linked to more favorable survival outcomes compared to late PTLD, may further explain the lack of association with mortality [35]. Collectively, these advances in treatment and monitoring likely attenuate any mortality differences attributable to EBV *D* serostatus.

Our study has several strengths: a large sample size from one of the largest transplant registries, representing the current transplant era, and the inclusion of patient‐level social determinants of health, such as insurance status. Although the OPTN database includes broad geographic information, such as state of residence and UNOS region, we did not have access to specific patient‐level geographic identifiers, such as ZIP codes, which are needed to link recipients to granular area‐level deprivation indices. Investigating geographic variations in PTLD incidence remains an important area for future research.

Several limitations should also be acknowledged. Selection bias may arise from excluding participants with missing EBV serostatus. However, variables relevant to PTLD, including CMV serostatus, race, and early‐treated rejection, did not demonstrate systematic differences between included and excluded participants, suggesting that baseline differences are unlikely to introduce significant bias in our main findings. Furthermore, our statistical approach accounted for other modest baseline differences. Misclassification of pre‐transplant EBV serostatus is a documented limitation of this registry. Potluri et al. demonstrated that inaccuracies in registry serostatus can lead to an underestimation of PTLD incidence and relative risk [33]. Additionally, a recent Danish study found that 55% of recipients classified as EBV R‐ were reclassified as EBV R+ after manual validation, due to misinterpretation of negative EBNA IgG alongside positive anti‐VCA IgG [34]. In our study, such misclassification could bias results toward the null, as EBV R‐ recipients misclassified as EBV R+ would inflate PTLD events in the EBV D + /R+ group, a pattern that was not observed. A further limitation was the absence of EBV DNAemia data; however, even if available, assay heterogeneity across centres would hinder harmonization [33]. As with other observational studies, ours is susceptible to unmeasured bias and residual confounding from factors such as allograft rejection, co‐infections, and genetic predisposition [18, 36, 37]. Registry‐based multicentre studies inherently involve less measurement control than single‐centre prospective studies, but this trade‐off was necessary to achieve adequate statistical power given the low incidence of PTLD and the rarity of EBV D‐ serostatus at the single‐centre level.

Ultimately, our study findings raise the question of whether EBV *D* serostatus should be considered in donor‐recipient matching in L/HLT beyond the traditional paradigm of avoiding EBV D+ organs for EBV R‐ candidates. For EBV R+ recipients, allocating EBV D‐ donor organs should be approached with caution, given the possible increased early PTLD risk noted in this group.

## 5 Conclusion

Although EBV D + /R− recipients carry the highest relative risk of PTLD, the overwhelming prevalence of EBV seropositivity among adult thoracic transplant recipients results in EBV R+ recipients comprising the majority of PTLD cases. Our findings identify EBV D‐/R + L/HLT recipients as a previously underrecognized group at increased risk of PTLD. Since routine serial monitoring of EBV DNAemia has shown limited predictive value in asymptomatic EBV R+ adults, prospective studies are needed to identify and develop targeted prevention strategies for this population.

## Author Contributions

**Khuloud Aldhaheri:** writing – review and editing, writing – original draft, project administration, methodology, investigation, formal analysis, data curation. **Robert C. Wright:** writing – review and editing, writing – original draft. **Stephen Lee:** writing – review and editing, data curation. **Mohsin Ali:** writing – review and editing, data curation. **Brian Clarke:** writing – review and editing. **Céline Bergeron:** writing – review and editing. **Allison Mah:** writing – review and editing. **Alissa Wright:** writing – review and editing. **Sara Belga:** writing – review and editing, supervision, project administration, methodology, investigation, formal analysis, data curation, conceptualization.

## Conflicts of Interest

The authors declare no conflicts of interest.

## Supporting information


Supporting File

## Data Availability

The data that support the findings of this study are available upon request from the Organ Procurement and Transplantation Network at https://optn.transplant.hrsa.gov/data/view-data-reports/request-data/.
