# Supplementary material for: Impact of Epstein‐Barr Virus Donor Serostatus on Post‐Transplant Mortality and Post‐Transplant Lymphoproliferative Disorder in Epstein‐Barr Virus Seropositive Thoracic Organ Transplant Recipients: Data from the Organ Procurement and Transplantation Network
Source: J Med Virol. 2026 Aug 24;98(8):e71117. doi: 10.1002/jmv.71117 (PMC13501425; doi:10.1002/jmv.71117)
Supplement: Supplementary file 1 [file JMV-98-e71117-s001.docx]

**Supplementary Material**

Supplement to: Aldhaheri K., Wright R., Lee S., et al. Impact of Epstein-Barr Virus Donor Serostatus on Post-Transplant Mortality and Post-Transplant Lymphoproliferative Disorder in Thoracic Organ Transplant Epstein-Barr Virus Seropositive Recipients: Data from the Organ Procurement and Transplantation Network.

**Index.**

**OPTN EBV anti-VCA IgG serostatus coding and distribution**…………………………..………3

**Definitions**…………………………………………………………………………………………...….4

**Comparison of analytical and excluded cohorts due to missing EBV serostatus**……….....7

**Cause of death stratified by organ transplant type and EBV D/R+ serostatus**…………….10

**Methods. Sensitivity Analyses: T-cell depleting therapy and triple maintenance immunosuppression**…………………………………………………………………………………11

**Methods. Exploratory Analyses: HLA mismatch and PTLD risk**………………………….....12

**Methods. Exploratory Analyses: PTLD classification and factors associated with early PTLD**……………………………………………………………………………………………………12

**Results. Table S3, Model A. T-cell depleting therapy**…………………………………….……..13

**Results. Table S3, Model B. Triple maintenance immunosuppression**.……………………..13

**Results. Table S4. HLA mismatch and PTLD risk**……………………………………………….15

**Results. Table S5. PTLD classification**……………………………………………………………16

**Results. Table S6. Factors associated with early PTLD**………………………………………..17

**The OPTN coding for EBV-VCA IgG serostatus and the distribution of non-interpretable results.**

The OPTN database records EBV anti-VCA IgG using the following standardized codes: Positive (P), Negative (N), Indeterminate/Equivocal (I), Not Done (ND), Pending (PD), Cannot Disclose (C), Unknown (U), and Not Reported (missing). Only Positive and Negative were considered interpretable. Below is the distribution of donor and recipients’ non-interpretable results among all OPTN records (n=143,898).

| **Code**  **(n=143,898)** | **Donor (n)** | **Recipient (n)** |
| --- | --- | --- |
| Not Reported (missing) | 41,988 | 22,557 |
| Not Done | 5,007 | 9,245 |
| Indeterminate/Equivocal | 461 | — |
| Unknown | 285 | 1,581 |
| Pending | 6 | — |
| Cannot Disclose | 5 | — |

**Definitions.**

***Exposure and EBV serostatus*.**

- Donor EBV serostatus: Determined by anti-VCA IgG testing as recorded on the OPTN serology form. For deceased donors, the deceased-donor EBV IgG field was used; for the small number of living donors, the living-donor EBV IgG field was used. Donors with a positive result were classified as EBV-seropositive (D+) and those with a negative result as EBV-seronegative (D-). Donors with indeterminate, pending, not-done, cannot-disclose, unknown, or missing results were excluded.
- Recipient EBV serostatus: Extracted from the OPTN recipient EBV status at transplant. The analytic cohort was restricted to EBV-seropositive recipients (R+); recipients recorded as seronegative, not done, unknown, or missing were excluded.
- Combined EBV D/R serostatus: Within the R+ cohort, combined serostatus was classified as D-/R+ or D+/R+, with donor serostatus as the exposure of interest.

***Outcomes.***

- PTLD: The primary outcome was de novo PTLD, reported on the OPTN post-transplant malignancy database, with the date of diagnosis.
- Early PTLD: PTLD diagnosed within the first year following transplantation (≤365 days), distinguishing early-onset.
- Patient survival: Death was defined from the OPTN composite patient status field. Survival time was the interval from transplant to death or, for survivors, to the last recorded follow-up.

***Recipient and donor characteristics.***

- Age: Donor and recipient ages at transplant (years) were analyzed as continuous variables (median, IQR). Recipient age was additionally categorized as 18–39, 40–59, and ≥60 years for descriptive purposes.
- Sex: Recorded as female or male, as submitted to UNOS/OPTN by the transplant centers.
- Race/ethnicity: Derived from the OPTN ethnicity category with Hispanic/non-Hispanic status, collapsed into a single mutually exclusive variable: non-Hispanic White, non-Hispanic Black, Hispanic or Latinx, and Other (including non-Hispanic Asian, American Indian/Alaska Native, Native Hawaiian/Pacific Islander, and multiracial).
- Insurance type: Primary payer at the time of transplant, categorized as Medicaid, Medicare, or private insurance; all remaining payer sources were grouped as Other.
- CMV D/R: Combined cytomegalovirus serostatus derived from donor and recipient CMV status at transplant, classified as D-/R-, D+/R-, D+/R+, or D-/R+ for descriptive purposes. In multivariable models, CMV exposure was represented as CMV mismatch (D+/R-) and recipient CMV-positive serostatus (R+).
- Pre-transplant mechanical assistance: ECMO and mechanical ventilation, each recorded at the time of transplant; a combined indicator denoted any pre-transplant mechanical assistance.
- Prior malignancy: Any previous recipient malignancy recorded by OPTN before transplantation.
- Waitlist time: Total days on the transplant waiting list, categorized as <1 year, 1–3 years, or ≥3 years.
- Organ type: Classified as lung, heart, or heart-lung. For stratified analyses, lung and heart-lung recipients were grouped together as a lung-containing thoracic allograft group and contrasted with isolated heart recipients.
- Transplant indication: Primary diagnosis at transplant. Descriptive data were limited to the three most prevalent indications per organ: for lung, COPD/emphysema, IPF, and cystic fibrosis; for heart, dilated cardiomyopathy (idiopathic, ischemic, or other); for heart-lung, pulmonary hypertension, IPF, and sarcoidosis.
- Early treated rejection: An acute rejection episode occurring before discharge from the index transplant admission that was treated with an anti-rejection agent (as distinct from untreated rejection or no rejection).
- HLA mismatch: The total number of donor–recipient HLA antigen mismatches across the HLA-A, HLA-B, and HLA-DR loci (OPTN-calculated, the sum of the per-locus mismatch levels). Each locus contributes 0–2 mismatches, yielding a total score ranging from 0 to 6. HLA mismatch variable in two forms: 1) continuous count of allele mismatches across HLA-A, HLA-B, and HLA-DR (range 0-6); 2) binary: low (0-2) vs high (≥3) mismatches. This variable was used in exploratory analysis.

***Immunosuppression.***

- T-cell depletion therapy: T Receipt of any lymphocyte-depleting agent (alemtuzumab, rabbit ATG, equine ATG, NRATG/NRATS, or muromonab-CD3) administered as induction or as treatment of rejection. Recipients receiving one or more of these agents were classified as exposed; those with immunosuppression data recorded but none of these agents were classified as unexposed; recipients with all relevant fields missing were coded as missing. This variable was used in sensitivity analyses.
- Induction immunosuppression: Categorized as;
  - IL-2 receptor antagonist (daclizumab, basiliximab, aldesleukin, denileukin diftitox).
  - Anti-thymocyte globulin (ATG).
  - Anti-CD20 (rituximab).
  - Anti-CD52 (alemtuzumab)
  - Other. The Other category pooled broad/common agents or agents with low contemporary use as induction agents: calcineurin inhibitors, mTOR inhibitors, antimetabolites, T-cell maturation inhibitors (deoxyspergualin), immunomodulators (leflunomide, methotrexate, anti-TNF agents), corticosteroids, cyclophosphamide, IL-6 and IL-1 receptor inhibitors, and miscellaneous monoclonal or investigational agents (anti-CD5, OKT3, OKT4, anti-ICAM-1, anti-LFA-1, fingolimod). Because of low frequency, declining use, and substantial missingness.
    - *Missing data: IL-2 receptor antagonist (n=20,027), ATG (n=19,755), anti-CD20 (n=47,755), anti-CD52 (n=20,027), Other (n=19,342)*
- Maintenance immunosuppression: Recorded by agent class prescribed at transplant;
  - Calcineurin inhibitors: all tacrolimus and cyclosporine formulations (n missing=1,792).
  - Tacrolimus: Prograf, Astagraf XL, extended-release, and generics (n missing=1,792).
  - Cyclosporine: Neoral, Sandimmune, SangCya, Gengraf, Eon, and generics (n missing=1,792).
  - Antimetabolites: mycophenolate mofetil, mycophenolic acid, azathioprine, brequinar sodium, mizoribine (n missing=2,639).
  - Mycophenolic acid: mycophenolate mofetil and delayed-release formulations (n missing=2,639).
  - Azathioprine: azathioprine/Imuran (n missing=2,639).
  - mTOR inhibitors: sirolimus and everolimus, including generics (n missing=3,037).
  - Corticosteroids — recorded under a single OPTN code (n missing=1,211).
  - Other: T-cell–depleting agents, IL-2 receptor blockers, immunomodulators, IL-6 inhibitors, belatacept, cyclophosphamide, and miscellaneous investigational/historical agents, pooled owing to low frequency and substantial missingness (n missing=20,292).
- Triple maintenance immunosuppression: Concurrent use of a calcineurin inhibitor, an antimetabolite, and corticosteroids at the time of transplant. Missing data for triple maintenance immunosuppression (n=724)

**Comparison of analytical and excluded cohorts due to missing EBV serostatus.**

**Aim**. To assess potential selection bias from excluding recipients with missing donor or recipient EBV anti-VCA IgG serostatus.

**Population**. The analytical cohort (n=49,485) was compared against recipients excluded due to missing EBV serostatus: 7,768 with missing donor anti-VCA IgG (EBV R+ recipients whose donor had a non-interpretable result (not reported, not done, indeterminant/equivocal, pending, cannot disclose, or unknown) and 4,733 with missing recipient anti-VCA IgG (interpretable donor serostatus, but recipient results are: not reported, not done, or unknown). Recipients excluded for other reasons (age <18 years, multiorgan other than HLT, or prior transplant) were not included. Additionally, recipients with both EBV D and R missing serostatus were not included.

**Analysis**: Donor and recipient age (continuous, years) reported as median (IQR); categorical variables as frequencies and proportions. Results in Table S1.

Supplementary **Table S1** presents baseline characteristics of the analytical cohort (stratified by EBV D-/R+ and D+/R+) alongside recipients excluded for missing donor serostatus (n=7,768) and missing recipient serostatus (n=4,733). Donor age was younger in D-/R+ (median 24, IQR 18-35) vs D+/R+ (median 32, IQR 23-44); recipient age was similar across groups (except mild imbalance in missing EBV D; median age 56 years vs 59 years). The excluded groups were broadly similar, though recipients with missing recipient serostatus had a higher proportion of ventilator support at transplant (7.1% vs 3.0-3.3%) and a higher proportion of heart transplants (66.9% vs 50.7-54.2%).

**Table S1.** Baseline characteristics of EBV R+ thoracic organ transplant recipients by donor serostatus (n=49,458) compared to excluded participants (n=13,244) with missing donor or recipient serostatus.

| **Factor**  **N (%)** | **EBV D-/R+**  n=3,248 | **EBV D+/R+**  n=46,210 | **Missing EBV D^1^, known EBV R+**  n=7,768 | **Missing EBV R^2^, known D**  n=4,733 |
| --- | --- | --- | --- | --- |
| **Age** | | | | |
| Donor, median (IQR)  Recipient, median (IQR)  **Recipient, categorical (years)**  18 – 39  40 – 59  ≥ 60 | 24.0 (18.0, 35.0)  59.0 (49.0, 65.0)  453 (13.9)  1312 (40.4)  1483 (45.7) | 32.0 (23.0, 44.0)  59.0 (50.0, 65.0)  5810 (12.6)  18884 (40.9)  21516 (46.6) | 29.0 (21.0–42.0)  56.0 (47.0–62.0)  1223 (15.7)  3729 (48.0)  2816 (36.3) | 32.0 (22.0–43.0)  57.0 (48.0–63.0)  688 (14.5)  2105 (44.5)  1940 (41.0) |
| **Male** | 2222 (68.4) | 30903 (66.9) | 5169 (66.5) | 3290 (69.5) |
| **Race, categorical** | | |  |  |
| White  Black  Hispanic or Latinx  Other | 2378 (73.2)  496 (15.3)  262 (8.1)  112 (3.4) | 33467 (72.4)  7450 (16.1)  3607 (7.8)  1686 (3.6) | 5825 (75.0)  1075 (13.8)  654 (8.4)  214 (2.8) | 3355 (70.9)  757 (16.0)  432 (9.1)  189 (4.0) |
| **Insurance type** | | |  |  |
| Medicaid  Medicare  Private | 335 (10.3)  1255 (38.6)  1536 (47.3) | 4559 (9.9)  18055 (39.1)  21825 (47.2) | 813 (10.5)  2498 (32.2)  4106 (52.9) | 484 (10.2)  1435 (30.3)  2650 (56.0) |
| **CMV D/R serostatus** | | |  |  |
| CMV D-/R-  CMV D+/R-  CMV D+/R+  CMV D-/R+ | 845 (26.0)  593 (18.3)  740 (22.8)  1070 (32.9) | 7485 (16.2)  12037 (26.1)  17111 (37.0)  9577 (20.7) | 1121 (14.4)  1846 (23.8)  3102 (39.9)  1699 (21.9) | 1035 (21.9)  1431 (30.2)  1464 (30.9)  803 (17.0) |
| **Mechanical assistance at the time of transplant** | | |  |  |
| ECMO  Ventilator | 92 (2.8)  99 (3.0) | 1547 (3.3)  1534 (3.3) | 83 (1.1)  217 (2.8) | 105 (2.2)  335 (7.1) |
| **Prior malignancy** | 266 (8.2) | 3978 (8.6) | 518 (6.7) | 360 (7.6) |
| **Time on transplant list, categorical (years)** | | |  |  |
| <1  1 – 3  ≥ 3 | 2763 (85.1)  384 (11.8)  101 (3.1) | 39447 (85.4)  5498 (11.9)  1265 (2.7) | 6336 (81.6)  1130 (14.6)  300 (3.9) | 4047 (85.5)  517 (10.9)  169 (3.6) |
| **Type of organ transplant** | | |  |  |
| Lung  Heart  Heart and lung | 1582 (48.7)  1646 (50.7)  20 (0.6) | 22328 (48.3)  23591 (51.1)  291 (0.6) | 3496 (45.0)  4213 (54.2)  59 (0.8) | 1518 (32.1)  3168 (66.9)  47 (1.0) |
| **Indication for lung transplant** | | |  |  |
| COPD  IPF  CF | 375 (23.7)  562 (35.5)  144 (9.1) | 4880 (21.9)  8260 (37.0)  1855 (8.31) | 1109 (31.7)  1092 (31.2)  398 (11.4) | 410 (27.0)  539 (35.5)  168 (11.1) |
| **Indication for heart transplant** | | |  |  |
| Dilated idiopathic cardiomyopathy  Dilated ischemic cardiomyopathy  Other dilated cardiomyopathy | 618 (37.6)  502 (30.5)  292 (17.7) | 8548 (36.2)  7398 (31.4)  4303 (18.2) | 1462 (34.7)  1613 (38.3)  579 (13.7) | 1104 (34.9)  1091 (24.4)  485 (15.3) |
| **Indication for heart/lung transplant** | | |  |  |
| PH  IPF  Sarcoidosis | 6 (30.0)  1 (5.0)  2 (10.0) | 111 (38.1)  28 (9.6)  19 (6.5) | 16 (27.1)  3 (5.1)  6 (10.2) | 17 (36.2)  3 (6.4)  2 (4.3) |
| **Early treated rejection** | 286 (8.8) | 4175 (9.0) | 632 (8.8) | 498 (10.5) |

Median (IQR) for continuous variables; n (%) for categorical variables.

^1^Missing donor EBV: EBV R+ recipients with non-interpretable donor anti-VCA IgG serostatus (Not Reported, Not Done, Indeterminate/Equivocal, Pending, Cannot Disclose, or Unknown)

^2^Missing recipient EBV anti-VCA IgG serostatus and known donor EBV anti-VCA IgG serostatus: recipients with non-interpretable recipient EBV serostatus (Not Reported, Not Done, or Unknown) but interpretable donor serostatus.

Abbreviations: CF: cystic fibrosis; CMV: cytomegalovirus; COPD: chronic obstructive pulmonary disease; D+: donor seropositive; D−: donor seronegative; D: donor serostatus; D/R: donor/recipient serostatus; D-/R-: donor-seronegative/recipient-seronegative; D+/R-: donor-seropositive/recipient-seronegative; D+/R+: donor-seropositive/recipient-seropositive; D-/R+: donor-seronegative/recipient-seropositive; EBV: Epstein-Barr virus; ECMO: extracorporeal membrane oxygenation; IPF: idiopathic pulmonary fibrosis; IQR: interquartile range; OPTN: Organ Procurement and Transplantation Network; PH: pulmonary hypertension; R+: recipient seropositive; R: recipient.

**Cause of death stratified by organ transplant type and EBV D/R+ serostatus.**

Supplementary **Table S2** presents causes of death stratified by organ type and donor EBV serostatus. Among lung recipients, pulmonary causes (19.1% vs 18.9%), infection (18.2% vs 18.3%), and graft failure (15.6% vs 16.8%) were the most common categories. Among heart recipients, cardiac causes (15.2% vs 16.6%) and infection (15.4% vs 16.4%) predominated. Malignancy as a cause of death was slightly higher in D-/R+ vs D+/R+ lung recipients (12.2% vs 10.2%). No meaningful differences in cause-of-death distribution by donor EBV serostatus were observed.

**Table S2**. Cause of death by donor EBV serostatus among EBV R+ thoracic organ

transplant recipients, stratified by organ transplant type.

|  | **Lung transplant** | | **Heart transplant** | | **Heart-lung transplant** | |
| --- | --- | --- | --- | --- | --- | --- |
|  | **EBV D-/R+**  n=784 | **EBV D+/R+**  n=11,623 | **EBV D-/R+**  n=473 | **EBV D+/R+**  n=7,160 | **EBV D-/R+**  n= 9 | **EBV D+/R+**  n=142 |
| Graft failure | 122 (15.6) | 1958 (16.8) | 45 (9.5) | 743 (10.4) | 0 (0.0) | 23 (16.2) |
| **Infection** | **143 (18.2)** | **2131 (18.3)** | **73 (15.4)** | **1171 (16.4)** | **1 (11.1)** | **18 (12.7)** |
| Bacterial | 69 (8.8) | 1100 (9.5) | 33 (7.0) | 549 (7.7) | 0 (0.0) | 8 (5.6) |
| CMV | 23 (2.9) | 190 (1.6) | 7 (1.5) | 77 (1.1) | 0 (0.0) | 1 (0.7) |
| Other viral | 25 (3.2) | 466 (4.0) | 17 (3.6) | 310 (4.3) | 0 (0.0) | 3 (2.1) |
| Fungal | 14 (1.8) | 168 (1.4) | 9 (1.9) | 112 (1.6) | 0 (0.0) | 1 (0.7) |
| Protozoal | 0 (0.0) | 1 (0.0) | 0 (0.0) | 2 (0.0) | 0 (0.0) | 0 (0.0) |
| Mixed or other | 12 (1.5) | 206 (1.8) | 7 (1.5) | 121 (1.7) | 1 (11.1) | 5 (3.5) |
| Cardiac | 48 (6.1) | 855 (7.4) | 72 (15.2) | 1189 (16.6) | 2 (22.2) | 20 (14.1) |
| Pulmonary | 150 (19.1) | 2193 (18.9) | 26 (5.5) | 470 (6.6) | 3 (33.3) | 19 (13.4) |
| Hematologic | 17 (2.2) | 158 (1.4) | 12 (2.5) | 120 (1.7) | 0 (0.0) | 5 (3.5) |
| Cerebrovascular | 24 (3.1) | 332 (2.9) | 26 (5.5) | 303 (4.2) | 1 (11.1) | 9 (6.3) |
| Malignancy | 96 (12.2) | 1184 (10.2) | 45 (9.5) | 673 (9.4) | 0 (0.0) | 5 (3.5) |
| Other | 58 (7.4) | 882 (7.6) | 63 (13.3) | 832 (11.6) | 1 (11.1) | 19 (13.4) |
| Unknown | 105 (13.4) | 1667 (14.3) | 86 (18.2) | 1397 (19.5) | 0 (0.0) | 19 (13.4) |
| Missing | 21 (2.7) | 263 (2.3) | 25 (5.3) | 262 (3.7) | 1 (11.1) | 5 (3.5) |

Data are presented as n (%). Percentages are calculated within each organ-type*donor EBV serostatus group.

Abbreviations: CMV: cytomegalovirus; D-/R+: donor-seronegative/recipient-seropositive; D+/R+: donor-seropositive/recipient-seropositive; EBV: Epstein-Barr virus; R+:recipient-seropositive.

**Methods: Sensitivity Analyses.**

1) **Adjustment for T-cell-depleting therapy sensitivity analysis.**

**Aim**. To assess whether the association between EBV donor serostatus and PTLD is confounded by T-cell-depleting therapy. **Population**. Complete-case analysis restricted to recipients with non-missing data for all covariates, including T-cell-depleting therapy  **Exposure**. EBV donor serostatus (D+ vs D-). **Outcome**. PTLD.  **Covariates.** EBV D serostatus, donor and recipient age (continuous, years), sex (female vs male), race (non-Hispanic White vs non-Hispanic Black), CMV D/R serostatus (CMV D+/R- and CMV R+ as separate indicators), early treated rejection (binary), and any T-cell-depleting therapy (binary, as defined in the Definitions section). **Analysis**. Lung/heart-lung and heart recipients were modeled separately. The primary multivariable Cox model was refitted with T-cell-depleting therapy added as a covariate. Results are presented in Table S3.

2) **Adjustment for triple maintenance immunosuppression sensitivity analysis.**

**Aim**. To assess whether the association between EBV donor serostatus and PTLD is confounded by receipt of triple maintenance immunosuppression at discharge.
**Population**. Complete-case analysis restricted to recipients with non-missing data for all covariates, including triple maintenance immunosuppression
**Exposure**. EBV donor serostatus (D+ vs D-). **Outcome**. PTLD.  **Covariates** EBV D serostatus, donor and recipient age (continuous, years), sex (female vs male), race (non-Hispanic White vs non-Hispanic Black), CMV D/R serostatus (CMV D+/R- and CMV R+ as separate indicators), early treated rejection (binary), and triple maintenance immunosuppression at discharge (binary, as defined in Definitions section). **Analysis** Lung/heart-lung and heart recipients were modeled separately. The primary multivariable Cox model was refitted with triple maintenance immunosuppression added as a covariate. Results are presented in Table S3.

**Methods: Exploratory Analyses.**

1) **Donor-recipient HLA mismatch and PTLD risk exploratory analysis.

   Aim**. To evaluate whether HLA mismatch is independently associated with PTLD risk after adjustment for EBV donor serostatus**.
   Population.** Complete-case analysis restricted to recipients with HLA typing available for all three loci (HLA-A, -B, -DR). **Exposure**. EBV donor serostatus (D+ vs D-). **Outcome**. PTLD.
   **Covariates.** EBV D serostatus, recipient and donor age (continuous, years), sex (female vs male), race (non-Hispanic White vs non-Hispanic Black), early treated rejection (binary), CMV D/R serostatus (CMV R+ and CMV D+/R- as separate indicators), and HLA mismatch. HLA mismatch was modelled in two forms:
1. Continuous: total allele mismatches across HLA-A, -B, and -DR (range 0-6).
2. Binary: low mismatch (0-2) versus high mismatch (≥3), using a threshold commonly adopted in thoracic transplant literature.

**Analysis**. Two separate multivariable Cox models were fit, one for each HLA mismatch specification. All covariates listed above were included in both models. Results are presented in Table S4.

2) **Logistic regression for early PTLD exploratory analysis.

   Aim**. To identify factors associated with early (≤365 days) versus late PTLD among recipients who developed PTLD during follow-up. **Population**: Recipients with confirmed PTLD and a recorded diagnosis date. **Exposure**. EBV donor serostatus (D+ vs D-). **Covariates**. Model A: EBV D serostatus and organ type (L/HLT vs HT). Model B: EBV D serostatus, organ type, and any T-cell-depleting therapy (as defined in Supplementary Appendix). **Outcome**. Early PTLD (≤365 days post-transplant).
   **Analysis**. Early vs late PTLD frequencies stratified by EBV D/R serostatus, presented in Table S5. Model A: Logistic regression adjusting for EBV D serostatus and organ type (n=663); results in Table S6. Model B: Logistic regression additionally adjusting for T-cell-depleting therapy. Sample size dropped to n=390 because T-cell depletion data were missing. Results in Table S6.

**Results: Sensitivity Analyses.**

Supplementary **Table S3** presents the sensitivity analyses.

**Model A** (T-cell-depleting therapy). Among LT/HLT recipients, EBV D+ remained independently associated with decreased hazard of PTLD (HR 0.46, 95% CI 0.31 – 0.68). T-cell-depleting therapy was not independently associated with PTLD (HR, 1.01; 95% CI, 0.67 – 1.52). Among HT recipients, EBV D+ showed no association (HR, 1.01; 95% CI, 0.53 – 1.93).

**Model B** (triple maintenance immunosuppression). EBV D+ was protective in LT/HLT (HR, 0.59; 95% CI, 0.42 – 0.83) but not in HT (HR, 1.20; 95% CI, 0.72 – 2.00). Triple maintenance immunosuppression was not associated with PTLD in either group.

**Table S3.** Multivariable Cox regression analyses for PTLD risk in EBV R+ thoracic organ transplant recipients, stratified by immunosuppression model.

|  | **Lung and heart-lung transplant** | **Heart transplant** |
| --- | --- | --- |
|  | **Adjusted HR (95% CI)** | **Adjusted HR (95% CI)** |
| **Model A – Adjusted for T-cell depleting therapy** | | |
| EBV (D+) | 0.46 (0.313 – 0.681) | 1.01 (0.527 – 1.925) |
| Donor age | 0.99 (0.986 – 1.004) | 0.99 (0.975 – 1.004) |
| Recipient age | 1.02 (1.007 – 1.032) | 1.02 (1.002 – 1.033) |
| Female sex | 0.76 (0.580 – 1.000) | 0.91 (0.624 – 1.339) |
| Black recipients  White recipients | 0.88 (0.484 – 1.592)  0.98 (0.636 – 1.509) | 0.96 (0.474 – 1.943)  1.47 (0.799 – 2.697) |
| T-cell depleting therapy | 1.01 (0.674 – 1.521) | 1.04 (0.752 – 1.440) |
| CMV D+/R-  CMV R+ | 1.41 (0.951 – 2.105)  1.09 (0.757 – 1.580) | 1.64 (0.938 – 2.873)  1.44 (0.851 – 2.446) |
| Early treated rejection | 1.25 (0.771 – 2.026) | 0.92 (0.539 – 1.581) |
| **Model B – Adjusted for maintenance triple immunosuppression** | | |
| EBV (D+) | 0.59 (0.421 – 0.825) | 1.20 (0.723 – 1.997) |
| Donor age | 1.00 (0.991 – 1.006) | 0.99 (0.983 – 1.004) |
| Recipient age | 1.02 (1.008 – 1.027) | 1.02 (1.010 – 1.033) |
| Female sex | 0.71 (0.573 – 0.893) | 0.74 (0.552 – 1.002) |
| Black recipients  White recipients | 0.68 (0.405 – 1.141)  0.94 (0.661 – 1.335) | 1.17 (0.690 – 1.972)  1.63 (1.033 – 2.571) |
| Triple immunosuppression | 0.99 (0.663 – 1.472) | 1.10 (0.713 – 1.703) |
| CMV D+/R-  CMV R+ | 1.20 (0.879 – 1.646)  1.04 (0.781 – 1.385) | 1.29 (0.897 – 1.853)  1.06 (0.755 – 1.485) |
| Early treated rejection | 1.41 (0.973 – 2.056) | 1.21 (0.840 – 1.743) |

Abbreviations: CI: confidence interval; CMV: cytomegalovirus; D+: donor-seropositive; D+/R-: donor-seropositive/recipient-seronegative; EBV: Epstein-Barr virus; HR: hazard ratio; PTLD: post-transplant lymphoproliferative disorder; R+: recipient-seropositive.

**Results: Exploratory Analyses.**

Supplementary **Table S4** presents the HLA mismatch analysis. Neither the continuous nor binary HLA mismatch measure was significantly associated with PTLD. Among LT/HLT: continuous HR 1.08 per mismatch (95% CI 0.98 – 1.19); high vs low HR 1.40 (95% CI 0.75 – 2.63). Among HT: continuous HR 1.02 (95% CI 0.91 – 1.15); high vs low HR 1.36 (95% CI 0.64 – 2.88). The protective effect of EBV D+ in LT/HLT remained significant after HLA adjustment (HR 0.63, 95% CI 0.44 – 0.90).

**Table S4.** Association of donor EBV serostatus and HLA mismatch with PTLD risk in multivariable Cox regression

|  | **Lung and heart-lung transplant** | **Heart transplant** |
| --- | --- | --- |
|  | **Adjusted HR (95% CI)** | **Adjusted HR (95% CI)** |
| EBV D+ | 0.63 (0.44 – 0.90) | 1.22 (0.71 – 2.10) |
| HLA mismatch (Continuous, per 1) | 1.08 (0.98 – 1.19) | 1.02 (0.91 – 1.15) |
| HLA mismatch (Binary, ≥3 vs 0–2) | 1.40 (0.75 – 2.63) | 1.36 (0.64 – 2.88) |
| Recipient age | 1.02 (1.01 – 1.03) | 1.02 (1.01 – 1.03) |
| Donor age | 1.00 (0.99 – 1.01) | 1.00 (0.98 – 1.01) |
| Female Sex | 0.73 (0.58 – 0.92) | 0.76 (0.55 – 1.04) |
| **Race** | | |
| Black | 0.69 (0.40 – 1.16) | 1.04 (0.61 –1 .78) |
| White | 0.92 (0.64 – 1.32) | 1.51 (0.95 – 2.39) |
| Early treated rejection | 1.50 (1.03 – 2.18) | 1.24 (0.84 – 1.82) |
| CMV R+ | 0.95 (0.71 – 1.28) | 1.07 (0.75 – 1.54) |
| CMV D+/R− | 1.10 (0.79 – 1.51) | 1.16(0.78 – 1.71) |

Abbreviations: CI: confidence interval; CMV: cytomegalovirus; D+: donor-seropositive; D+/R-: donor-seropositive/recipient-seronegative; EBV: Epstein-Barr virus; HLA: human leukocyte antigen; HR: hazard ratio; R+: recipient-seropositive; TOT: thoracic organ transplant; PTLD: post-transplant lymphoproliferative disorder

Supplementary **Table S5** presents PTLD timing by organ and donor serostatus. Among LT/HLT, a significantly higher proportion of PTLD cases were early in D-/R+ (48.7%) vs D+/R+ (21.9%). Among HT, early PTLD was 6.2% in D-/R+ vs 10.9% in D+/R+. The excess PTLD risk with D- status in LT/HLT is concentrated in the first post-transplant year.

**Table S5**. Early vs late PTLD classification by EBV D/R serostatus among EBV-seropositive thoracic organ transplant recipients

| **PTLD Classification**^1^  **N (%)** | **Lung and heart-lung transplant** | | **Heart transplant** | |
| --- | --- | --- | --- | --- |
|  | **EBV D-/R+** | **EBV D+/R+** | **EBV D-/R+** | **EBV D+/R+** |
| Early PTLD^2^ | 19 (48.7) | 73 (21.9) | 1 (6.2) | 30 (10.9) |
| Late PTLD^3^ | 20 (51.3) | 261 (78.1) | 15 (93.8) | 244 (89.1) |

^1^The denominator for calculating internal proportions of early and late PTLD is the total cohort of patients with PTLD ^2^Early PTLD is defined as occurring the first year post-transplant

^3^Late PTLD is defined as occurring 1 or more years post-transplant

Abbreviations: D-/R+: donor-seronegative/recipient-seropositive, D/R: donor-serostatus/recipient-serostatus; D+/R+: donor-seropositive/recipient-seropositive, EBV: Epstein-Barr virus, PTLD: post-transplant lymphoproliferative disorder, R+: seropositive recipients.

Supplementary **Table S6** presents logistic regression results. EBV D+ was associated with lower odds of early PTLD (Model A: aOR, 0.40; 95% CI, 0.22 – 0.73; Model B: aOR, 0.40; 95% CI, 0.22-0.74). LT/HLT was associated with higher odds of early PTLD vs HT (Model A: aOR, 2.62; 95% CI, 1.68 – 4.08; Model B aOR, 2.58; 95% CI, 1.62 – 4.10). T-cell depletion was not significantly associated with early PTLD (aOR, 0.89; 95% CI, 0.51 – 1.55).

**Table S6.** Logistic regression analysis of factors associated with early PTLD among EBV R+ thoracic organ transplant recipients

| **Variable** | **aOR (95% CI)** |
| --- | --- |
| **Model A (n = 663)^1^** | |
| EBV D+ | 0.40 (0.22 – 0.73) |
| Lung or heart-lung transplant | 2.62 (1.68 – 4.08) |
| **Model B (n = 390)^2^** | |
| EBV D+ | 0.40 (0.22 – 0.74) |
| Lung or heart-lung transplant | 2.58 (1.62 – 4.10) |
| T-cell depletion | 0.89 (0.51 – 1.55) |

^1^ Model A includes all PTLD cases (n = 663).

^2^ Model B restricts to PTLD cases with complete T-cell depletion data (n = 390).

Abbreviations: PTLD: post-transplant lymphoproliferative disorder; aOR: adjusted odds ratio; CI: confidence interval; EBV: Epstein-Barr virus; D+: donor seropositive.
